# Advancing Psychiatric Curricula Through a Diverse Lens

**DOI:** 10.1007/s40596-022-01689-y

**Published:** 2022-08-05

**Authors:** Rahn K. Bailey, Ronja R. Bodola, Ayush Arora

**Affiliations:** Louisiana State University Health Sciences Center, New Orleans, LA USA

To the Editor:

In January 2021, the American Psychiatric Association (APA) issued an apology to Black, Indigenous, and People of Color, which covered the organization’s past silence on issues that affected marginalized ethnic and gender-based minority groups. This silence has contributed to reduced quality of care [1]. This is unacceptable. A current lack of emphasis on social determinants of health in preclinical curricula across medical schools perpetuates this issue [2]. Even when present in curricula, often the discussion does not revolve around society’s inequitable practices exacerbating patient health outcomes [3]. Therefore, to deliver impactful change for the next generation of psychiatrists, it is important to make additions and changes to psychiatry curricula and, ideally, medical school curricula nationwide.

Though there have been significant points raised regarding the inclusion of social determinants of health into medical education, including the integration of critical race theory (CRT) [4], there has not been a clear-cut proposal for how best to include these ideas into a cohesive educational model. It will not do to simply introduce concepts; we have to teach them. Just recently, the updated outline for the cultural formulation in the DSM-5-TR [5] reminds us that race is a social construct. Statements like these, or tools like the cultural formulation interview, are important. However, they fall short in helping learners understand what is meant by social constructedness and its systemic underpinnings that perpetuate health disparities.

Based on our own curricula renewal to address these topics, we argue that the so-called critical medical humanities [6] can play a decisive role in teaching the students about the barriers to equitable outcomes for many patients: Social and cultural critique and constructivist thought are at the core of many humanist approaches, and critical medical humanities focus on critical theory (including CRT), gender studies, biopolitics, and disability studies to examine the power structures, systemic patterns, and issues of sociocultural identities that lead to health care inequities.

This is not to say that we should teach abstract humanist theory to medical students. On the contrary, it is key to make those approaches as relevant and concrete as possible by focusing on specific core concepts that make the issues tangible. One example from CRT is intersectionality, a concept that is rooted in feminist criticism and ethics of care. It illustrates issues of intersecting social identities that may impact health disparities in a way that every learner can understand. It includes aspects of race and ethnicity but is not limited to them, thus exceeding an anti-racist lens. We combine these concepts with other interdisciplinary, humanist methodologies such as reflective writing and storytelling to help learners gain insight into diverse lived experiences. Thus, our curriculum renewal efforts combine three major trends in academic medicine: intersectionality, narrative medicine, and medical humanities.

Due to varying curriculum structures across medical schools, we understand that adding an entire course is not always possible, nor necessarily desirable: We argue that we need to include critical concepts and methodologies into existing lessons, for instance, while teaching core psychiatric practices such as interviewing, history taking, or understanding the cultural sections in the DSM-5, to name just a few. To that end, our department developed a number of undergraduate and graduate medical education modules that center around intersectionality. One example is an asynchronous lecture and reflective writing exercises titled “Ethics, Identities and Mental Health,” which was integrated into our 6-week clerkship. It built on already existing psychiatric ethics modules, so no new course was added. Preliminary results of analyzing students’ reflective pieces (*N* = 138) suggest an increasing awareness of diversity and systemic issues over the course of the module. Furthermore, our school revised the first-year clinical skills course. They added social backgrounds to existing clinical vignettes of patients with small group discussions that not only consider how these backgrounds advanced the disease process but how systemic factors further contributed.

In an ideal world, there would be a mandatory “Diversity and Social Determinants of Health” course in the first year of medical school that is as integrated as the above examples. This course would explore the social and systemic factors leading to adverse outcomes for underserved and minority patient groups and approach medical education with a diverse lens. Medical students would not only be taught by academicians and clinicians but also by community health care stakeholders. They could discuss the pertinent social factors affecting these patients’ care as well as systemic factors that may negatively impact these patients’ livelihoods. The assignments would ideally allow a space for students to critically think and elaborate on their thoughts through reflective writing assignments as opposed to traditional multiple-choice exams. Through verbal and written exploration of issues in health care, medical students will be better equipped to apply lessons learned in future practice and become better advocates for their patients’ health.

Another facet of the course could be an analysis of how disasters can impact health care delivery among patients of color and those with lower incomes. For instance, throughout the COVID-19 pandemic, we have all witnessed the increased incidence of disease and death among these underserved patients, especially when considering psychiatric disorders. An analysis of the COVID-19 pandemic’s social impact, which worsened mental health for many people, should be explained in medical school, as future doctors need to understand how environmental stressors contribute to psychiatric disease. Achieving this goal relies on medical students learning to recognize the underlying power structures that contribute to difficulties in health care access for many patients.

We envision improved patient outcomes by including an integrative, interdisciplinary “Social Determinants of Health” course or comparable content into medical school and psychiatric curricula. We have an opportunity to teach how sociopolitical factors affect quality of care and to develop inclusive ideals among the next generation of doctors. Incorporating critical medical humanities into medical school curricula has the potential to force an important evaluation of existing practices by providing the critical concepts and frameworks that can impact our thought styles and pave the way for a more equitable future.
